# Adolescent chemogenetic activation of dopaminergic neurons leads to reversible decreases in amphetamine-induced stereotypic behavior

**DOI:** 10.1186/s13041-024-01110-9

**Published:** 2024-06-11

**Authors:** Muhammad O. Chohan, Amy B. Lewandowski, Rebecca N. Siegel, Kally C. O’Reilly, Jeremy Veenstra-VanderWeele

**Affiliations:** 1 Department of Psychiatry, Columbia University Medical Center, New York, NY 10032 USA; 2 New York State Psychiatric Institute, New York, NY 10032 USA; 3 Department of Psychological & Brain Sciences, Washington University in St. Louis, St. Louis, MO 63130 USA

**Keywords:** Chemogenetics, hM3D(Gq), Dopamine, EAAT3, Adolescence, Amphetamine, Locomotion, Stereotypic behavior

## Abstract

Chronic perturbations of neuronal activity can evoke homeostatic and new setpoints for neurotransmission. Using chemogenetics to probe the relationship between neuronal cell types and behavior, we recently found reversible decreases in dopamine (DA) transmission, basal behavior, and amphetamine (AMPH) response following repeated stimulation of DA neurons in adult mice. It is unclear, however, whether altering DA neuronal activity via chemogenetics early in development leads to behavioral phenotypes that are reversible, as alterations of neuronal activity during developmentally sensitive periods might be expected to induce persistent effects on behavior. To examine the impact of developmental perturbation of DA neuron activity on basal and AMPH behavior, we expressed excitatory hM3D(Gq) in postnatal DA neurons in TH-Cre and WT mice. Basal and CNO- or AMPH-induced locomotion and stereotypy was evaluated in a longitudinal design, with clozapine N-oxide (CNO, 1.0 mg/kg) administered across adolescence (postnatal days 15–47). Repeated CNO administration did not impact basal behavior and only minimally reduced AMPH-induced hyperlocomotor response in adolescent TH-Cre^hM3Dq^ mice relative to WT^hM3Dq^ littermate controls. Following repeated CNO administration, however, AMPH-induced stereotypic behavior robustly decreased in adolescent TH-Cre^hM3Dq^ mice relative to controls. A two-month CNO washout period rescued the diminished AMPH-induced stereotypic behavior. Our findings indicate that the homeostatic compensations that take place in response to chronic hM3D(Gq) stimulation during adolescence are temporary and are dependent on ongoing chemogenetic stimulation.

**Supplementary Information:**

The online version contains supplementary material available at 10.1186/s13041-024-01110-9.

## Main text

We previously found unexpected decreases in amphetamine (AMPH)-induced behavior and dopamine (DA) transmission in mice with loss of the neuronal glutamate transporter EAAT3 [1], and reciprocal increases in behavior and transmission in mice with DA neuron-conditional EAAT3 overexpression [2]. Interestingly, adult overexpression was not sufficient to alter behavior in EAAT3-overexpressing animals [2], indicating that alterations in DA transmission during developmentally sensitive periods [3] are necessary for the effects of EAAT3 overexpression on AMPH response. This would also be consistent with prior research showing increased motor activity and decreased AMPH response following chronic inhibition and excitation of DA neurons, respectively, during development, but not in adult animals [4–6].

To directly test the hypothesis that AMPH-induced behavior can be influenced by chronically altering DA neuron activity, we previously evaluated the impact of repeated chemogenetic activation of DA neurons in adult mice, finding decreased baseline locomotion, AMPH response, and DA transmission [7]. The diminished basal and AMPH-induced behaviors were restored after stopping clozapine N-oxide (CNO) treatment, indicating that the compensations in DA transmission that occur in response to chronic chemogenetic stimulation in adulthood are dependent on concurrent stimulation.

**Fig. 1:**
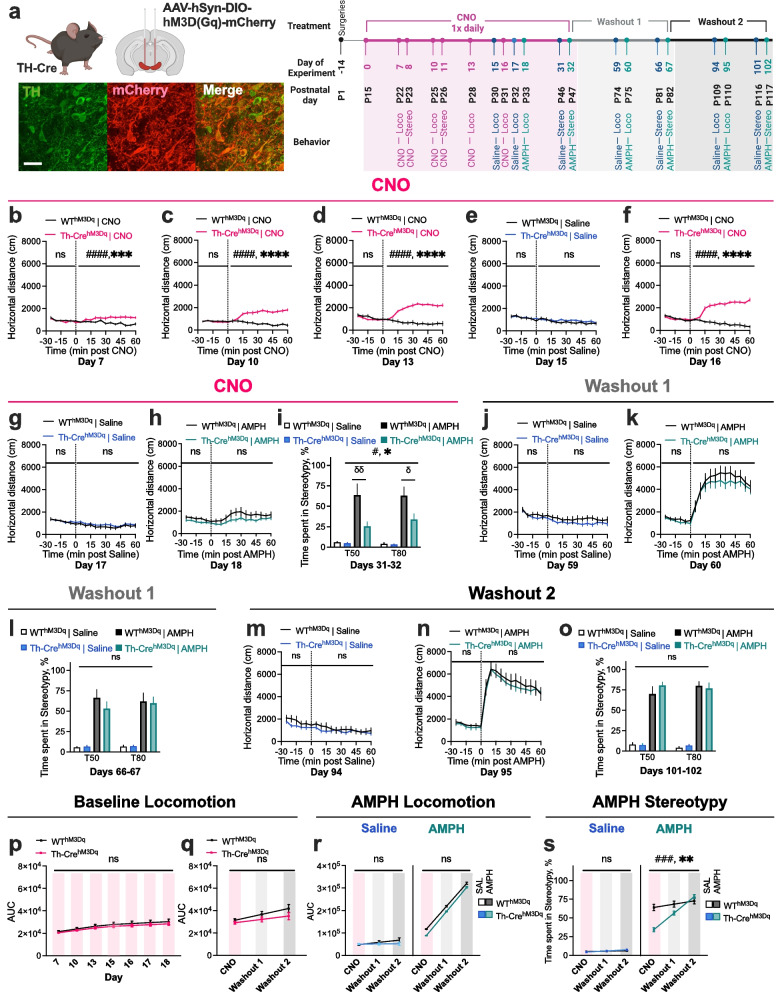
Repeated chemogenetic stimulation of DA neurons during adolescence induces reversible decreases in AMPH-induced stereotypic behavior. **a** *(Left)* Schematic of viral injection and representative sections from the midbrain region of TH-Cre^hM3Dq^ mice. Scale bar: 50 um. *(Right)* Experimental timeline. Surgeries were conducted in postnatal day 1 (P1) pups. CNO (1.0 mg/kg IP, 1 × daily) was administered from P15 to P47 (Experimental Days 0–32 = ‘CNO phase’). CNO, saline, and AMPH-induced locomotion and stereotypy were tested during the CNO phase. Mice were again tested one month and two months after stopping CNO during Washout phases 1 and 2. **b-d, f** TH-Cre^hM3D(Gq)^ mice show progressive increases in CNO-induced hyperlocomotion from Days 7–18. Repeated CNO does not impact baseline (pre-injection) locomotion in TH-Cre^hM3D(Gq)^ mice. **e, g** Locomotor activity after IP saline is unaltered in TH-Cre^hM3D(Gq)^ mice. **h** TH-Cre^hM3D(Gq)^ mice show decreased AMPH (3.0 mg/kg)-induced hyperlocomotor response relative to WT^hM3D(Gq)^ mice after repeated CNO via curve fit analysis, but not via 2-way RM ANOVA. (**i**) TH-Cre^hM3D(Gq)^ mice show diminished AMPH (8.0 mg/kg)-induced stereotypic behavior in CNO phase via three-way ANOVA. **j, k, m, n** No genotype difference in baseline and AMPH-induced locomotion is observed after one month and two months of CNO washout. **l, o** No genotype difference in AMPH-induced stereotypy is observed after one month and two months of washout. **p-r** No genotype difference in baseline and AMPH-induced locomotor response is observed across CNO and Washout phases via Linear Mixed Model (LMM). **s** TH-Cre^hM3D(Gq)^ mice show decreased AMPH-induced stereotypic behavior in CNO phase and recovery of AMPH response after CNO washout via LMM. (*N* = 13 TH-Cre^hM3Dq^ and 10 WT^hM3Dq^ mice. Two-way treatment x genotype interaction, ^*###*^*P* < 0.001, ^*#*^*P* < 0.05; genotype effect, **P < 0.01, *P < 0.05; Holm-Sidaks’s multiple comparisons, ^σσ^*P* < 0.01, ^σ^*P* < 0.05. ^ns^*P* = not significant. Also see Additional Figs. S1-3 and Additional Table 1.)

Based on our EAAT3 [1, 2] findings, we hypothesized that repeated adolescent stimulation of DA neurons will lead to diminished baseline activity and AMPH response. Adult chemogenetic findings [7], coupled with our data showing that restoration of EAAT3 expression in adulthood reversed changes in AMPH response [1, 2], led us to further hypothesize that stopping CNO will lead to restoration of behavior. An alternative hypothesis, based on prior developmental work [3–6], would be that perturbation of DA neurotransmission during adolescence could lead to enduring impact on baseline and AMPH behavior. We employed a longitudinal study design in 13 TH-Cre^hM3Dq^ and 10 WT^hM3Dq^ mice to evaluate the impact of adolescent chemogenetic stimulation on basal activity and AMPH responses. To evaluate for persistent impact, basal activity and AMPH behavior was evaluated again in adult mice one and two months after stopping CNO (Fig. 1a and Additional Methods).

One week after beginning daily intraperitoneal (IP) 1.0 mg/kg CNO treatment, open field activity was modestly increased following CNO in TH-Cre^hM3D(Gq)^ mice on postnatal day 22 (P22) (Fig. 1b; See Additional Table 1 for statistics). Between P22 and P31, TH-Cre^hM3D(Gq)^ mice showed cumulative increases in CNO-induced hyperlocomotor response (Figs. 1b-d, f, and Additional Figs. 1–2).

Unlike in adult animals [7], repeated CNO administration in adolescents did not impact baseline locomotion, measured over the 30 min period immediately prior to IP injection (Fig. 1b-h) or in 60 min following IP saline injection by two-way RM ANOVA (Fig. 1e, g), in TH-Cre^hM3D(Gq)^ mice, with decreased locomotion observed only at P33 by curve-fit analysis (*P* = 0.0111). Following repeated CNO, no genotype difference was observed in hyperlocomotor response to IP AMPH (3.0 mg/kg) by two-way RM ANOVA (Fig. 1h), however, curve-fit analysis revealed a significant main effect of genotype (*P* < 0.0001), and area under the curve (AUC) analysis showed a significant treatment-by-genotype interaction (*P* = 0.0127) (Additional Fig. 1), indicating modestly decreased AMPH-induced locomotion in TH-Cre^hM3D(Gq)^ mice.

Examination of stereotypic behavior following CNO showed no differences between adolescent TH-Cre^hM3D(Gq)^ mice and WT^hM3D(Gq)^ controls (Additional Fig. 3). Administering high-dose (8.0 mg/kg) AMPH to animals exposed to repeated CNO, however, revealed a significant decrease in stereotypic response in TH-Cre^hM3D(Gq)^ mice (Fig. 1i). A three-way ANOVA revealed significant effects of AMPH treatment (*P* < 0.0001) and genotype (*P* = 0.01), and an interaction between treatment and genotype (*P* = 0.0210). Holm-Sidak’s multiple comparisons revealed significantly lowered stereotypy at 50-min (T50) (*P* = 0.0048) and 80-min (T80) (*P* = 0.0449) post-AMPH injection in TH-Cre^hM3D(Gq)^ mice compared to controls.

Following one or two months of CNO washout, no genotype difference was observed in baseline locomotion (Fig. 1j, m) or AMPH-induced hyperlocomotion (Fig. 1k, n) by two-way RM ANOVA, curve-fit, or area under the curve (AUC) analysis (Additional Fig. 1). Further, three-way ANOVA and Linear Mixed Model (LMM) [8] analysis revealed no genotype differences across CNO and Washout phases (Fig. 1p-r and Additional Fig. 1). After one or two months of CNO washout, genotype effects were no longer observed for stereotypic response to high-dose AMPH (Fig. 1l, o). A three-way ANOVA revealed that there were significant main effects of CNO phase (*P* = 0.0007) and AMPH treatment (*P* < 0.0001), and phase-by-treatment interaction (*P* = 0.0055), phase-by-genotype interaction (*P* = 0.0253), and trend-level phase-by-treatment-by-genotype interaction (*P* = 0.0571). Holm-Sidak’s multiple comparisons showed that TH-Cre^hM3D(Gq)^ mice displayed lower AMPH-induced stereotypy in the repeated CNO phase (*P* = 0.0177) but showed comparable levels of stereotypy to WT^hM3D(Gq)^ controls after one month (*P* = 0.99), and two months (*P* > 0.99) of washout. Based on estimated fixed effects via LMM, there were no significant main effects of genotype (*P* = 0.557), or phase-by-genotype interaction (*P* = 0.428) following saline challenge; however, there were significant main effects of genotype (*P* = 0.007), and interaction between phase and genotype (*P* = 0.012) following AMPH challenge (Fig. 1s).

In partial agreement with our hypothesis, we found that repeated chemogenetic stimulation of DA neurons during adolescence leads to decreased AMPH-induced stereotypy in late-adolescent mice. The decreased stereotypic response to AMPH was restored after CNO washout, indicating that maintenance of this effect requires continuing DA neuron stimulation. A caveat for our results is that sensitization could potentially contribute to the rescue of AMPH behavior. Future studies could evaluate behavior in naïve cohorts during washout phase. Overall, our findings partially align with our recent findings that show reversible decreases in AMPH behavior in adult CNO-treated TH-Cre^hM3D(Gq)^ mice [7], and suggest that changes in AMPH response that follow long-term chemogenetic stimulation generalize across adulthood and adolescence.

With regard to downstream correlates of diminished AMPH-induced stereotypy in TH-CrehM3D(Gq) mice, our recent demonstration of a positive correlation between AMPH-induced stereotypic behavior and cFos expression in ventral medial striatum DA D1-receptor expressing medium spiny neurons (D1-MSNs) [9], combined with evidence in prior work of blunted D1-MSN postnatal maturation in Pitx3KO mice [10], might lead us to expect diminished D1-MSN activity, particularly in the ventral medial striatum region, in TH-CrehM3D(Gq) mice, although D2-MSN activity may also be impacted based on its well-known role in the maintenance of repetitive behaviors [11]. Future experiments utilizing in vivo recordings in the dorsal and ventral striatum would be necessary to establish the precise neuronal correlates of diminished AMPH-induced stereotypic response.

The diminished stereotypic response to high-dose AMPH was not paralleled by effects of repeated chemogenetic stimulation on baseline locomotion and was associated with only minimally decreased AMPH-induced hyperlocomotion in early-adolescent mice. This may reflect an immature DA system that is characterized by less overall locomotor response to AMPH [10, 12–15]. Because of the substantially lower DA release capacity and DAT expression during early adolescence [12, 16], as well as the DA-independent maturation of MSNs prior to P18 [10], it is conceivable that DREADD activation would only minimally impact stereotypic response to high-dose AMPH in early-adolescent mice, at least prior to P18. Future studies could evaluate the impact on AMPH-induced locomotion in late adolescence, when expression of psychomotor behavior is at adult levels, as evidenced by our stereotypy data. However, the lack of a genotype difference in stereotypy after CNO washout suggests that any changes in locomotion that are found in late adolescence would be dependent on concurrent chemogenetic stimulation.

While our experimental paradigm does not allow us to fully rule out a role for context conditioning in the progressive increases in CNO-induced hyperlocomotor response, for several reasons we believe that the ongoing maturation of the DA system during this time period is what underlies this behavior. First, context conditioning would be predicted to lead to increased anticipatory activity [17, 18], but we find no changes in pre-injection locomotion. Second, no conditioned response to saline injections, a well-known phenomenon of associative learning [19, 20], is observed in TH-CrehM3Dq mice. Third, conditioning would be expected to increase AMPH-induced hyperlocomotor response [21, 22], but we find the opposite in TH-CrehM3Dq mice. Fourth, it is well-established that the DA system exhibits protracted maturation, with locomotor-inducing D1-MSNs acquiring maturation at P28 (Day 13 of CNO exposure in our study) [10] and psychomotor activity modulating mesocortical DA projections not acquiring maturation until early adulthood [13–15]. In support of this, we found ~ threefold lower locomotor activation after CNO and AMPH in adolescent TH-CrehM3Dq mice compared to their adult counterparts, mirroring the ~ threefold lower DA transmission and AMPH sensitivity that is present in early-adolescence relative to adulthood [10, 12]. Finally, repeated CNO in adult mice does not lead to cumulative increases in CNO-induced hyperlocomotor response [7].

Recently, Salesse and colleagues reported increased baseline locomotion and stereotypy following chronic (2 × daily, 0.5 mg/kg CNO injections) hM4DG(i)-mediated inhibition in DAT-Cre mice during development (P14-30) [4]. Notably, G(q) and G(i) are expected to induce diverse homeostatic changes upon chronic stimulation due to activation of disparate intracellular cascades. Further, in contrast to the TH-Cre line, the DAT-Cre line has been reported to display basal hyperactivity and reduced AMPH sensitivity [23, 24], potentially confounding interpretation of these data. It will be important to evaluate the impact of inhibition of DA neurons on AMPH behavior in future studies, to test whether reciprocal changes are induced in AMPH response.

The paradoxical decreases in AMPH-induced stereotypic behavior in adolescent mice likely reflect homeostatic adaptations in DA neurotransmission, as we saw for EAAT3 and adult CNO-treated TH-Cre^hM3D(Gq)^ mice [1, 2, 7]. Future studies could evaluate the impact on DA neuron activity to test whether mechanisms that underlie diminished AMPH response in adult animals also extend to adolescence. Together, our convergent adult [7] and adolescent findings underscore the need to factor in homeostatic adaptations when interpreting results of chronic chemogenetic experiments.

### Supplementary Information

Supplementary Material 1.

## Data Availability

The datasets used and/or analyzed during the current study are available from the corresponding author on reasonable request.
