# Supplementary material for: Adolescent chemogenetic activation of dopaminergic neurons leads to reversible decreases in amphetamine-induced stereotypic behavior
Source: Mol Brain. 2024 Jun 11;17:36. doi: 10.1186/s13041-024-01110-9 (PMC11165814; doi:10.1186/s13041-024-01110-9)
Supplement: Supplementary file 1 — Supplementary Material 1. [file 13041_2024_1110_MOESM1_ESM.docx]

**Additional file**

**Adolescent chemogenetic activation of dopaminergic neurons leads to reversible decreases in amphetamine-induced stereotypic behavior**

Muhammad O. Chohan^1,2*^, Amy B. Lewandowski^2,3^, Rebecca N. Siegel^1,2^, Kally C. O’Reilly^1,2^, Jeremy Veenstra-VanderWeele^1,2^

^1^Department of Psychiatry, Columbia University Medical Center, New York, NY 10032, USA; ^2^New York State Psychiatric Institute, New York, NY 10032, USA; ^3^Department of Psychological & Brain Sciences, Washington University in St. Louis, St. Louis, MO 63130, USA

***Correspondence:**

Muhammad O. Chohan ([muhammad.chohan@nyspi.columbia.edu](mailto:muhammad.chohan@nyspi.columbia.edu))

**This Additional file includes:**

Additional Figures 1-3

Additional Table 1

Additional Methods

**Additional Figures**

**Additional Fig. 1 Unaltered baseline locomotion and modestly decreased AMPH-induced locomotion in CNO treated early adolescent TH-Cre^hM3D(Gq)^ mice compared to WT^hM3D(Gq)^ controls: area under the curve (AUC) analysis**

**(a-c)** AUC analysis of baseline (pre-injection) locomotor activity after repeated CNO administration in TH-Cre^hM3D(Gq)^ mice and WT^hM3D(Gq)^ controls during CNO phase (P22-33) **(a)**, following one month of CNO washout (P74-75) **(b)**, and following two months of CNO washout (P109-110) **(c)**. There were no significant effects of day and genotype, or interaction between day and genotype on pre-injection baseline locomotor activity in the CNO phase based on two-way RM ANOVA (day x genotype interaction *F* (4, 84) = 0.4763, ^ns^*P* = 0.7530, genotype *F* (1, 21) = 0.3883, *P* = 0.5399, **a, *left***). Further, no genotype difference in pre-injection baseline locomotion was observed on saline or AMPH test days (P32-33) (day x genotype interaction *F* (1, 20) = 10.15, ^##^*P* = 0.0046, day *F* (1, 20) = 0.0078, *P* = 0.9375, genotype *F* (1, 21) = 0.6314, ^ns^*P* = 0.4375, **a, *right***). Finally, no significant effect of genotype on baseline locomotion was observed following one month of CNO washout (P74-75) (interaction *F* (1, 21) = 0.0002, ^ns^*P* = 0.9881, genotype *F* (1, 21) = 1.013, *P* = 0.3256, **b**); and following two months of washout (P109-110) (interaction *F* = 1.674, ^ns^*P* = 0.2098, genotype *F* = 1.180, *P* = 0.2897, **c**).

**(d-e)** AUC analysis of post-injection locomotor activity during CNO phase (P22-33) **(d)**, following one month of CNO washout (P74-75) **(e)**, and following two months of washout (P109-110) **(f)**. Analysis of post-injection locomotor activity showed progressive increases in CNO-induced hyperlocomotor response in TH-Cre^hM3D(Gq)^ mice compared to WT^hM3D(Gq)^ controls between P22 and P31 by two-way RM ANOVA (day x genotype interaction *F* (4, 84) = 31.14, ^####^*P* < 0.0001, genotype *F* (1, 21) = 78.10, *****P* < 0.0001, day *F* (4, 84) = 20.23, *P* < 0.0001; Holm-Sidak’s multiple comparisons: TH-Cre^hM3D(Gq)^ vs WT^hM3D(Gq)^ Day 7, ^δδ^*P* = 0.0049; Day 10, ^δδδδ^*P* < 0.0001; Day 13, ^δδδδ^*P* < 0.0001; Day 16, ^δδδδ^*P* < 0.0001; TH-Cre^hM3D(Gq)^ Day 7 vs 10, *P* = 0.0011; Day 10 vs 13, *P* < 0.0001; Day 13 vs 16, *P* = 0.0556; **d, *left***). No genotype difference in AUC was observed on the saline test day (P15) (Holm-Sidak’s multiple comparisons, Day 15, ^ns^*P* = 0.3478, **d, *left***). A significant treatment-by-genotype interaction was observed on the hyperlocomotor response after AMPH administration versus saline in the CNO phase, as well as a significant main effect of treatment (P32-33) (treatment x genotype interaction *F* (1, 20) = 7.500, ^#^*P* = 0.0127, treatment *F* (1, 20) = 20.23, *P* < 0.0001, genotype *F* (1, 21) = 0.5625, ^ns^*P* = 0.4616; Holm-Sidak’s multiple comparisons: saline, *P* = 0.4906; AMPH, *P* = 0.1073, **d, *right***). No significant difference in AMPH-induced hyperlocomotor response between TH-Cre^hM3D(Gq)^ mice and WT^hM3D(Gq)^ controls was observed at one month of CNO washout (P74-75) (interaction *F* = 0.0222, ^ns^*P* = 0.8831, genotype *F* = 1.115, *P* = 0.3029, **e**), or two months of washout (P109-110) (interaction *F* = 0.0816, ^ns^*P* = 0.7780, genotype *F* = 0.5100, *P* = 0.4830, **f**).

Notably, the AMPH-induced hyperlocomotor response was age-dependent, with only a minimal response observed in the CNO phase at P33 compared to responses in Washout phase 1 at P75 and Washout phase 2 at P110 (three-way ANOVA, CNO phase x AMPH treatment interaction *F* (2, 40) = 43.66, *P* < 0.0001, phase x genotype interaction *F* (2, 42) = 0.1986, *P* = 0.8206, treatment x genotype interaction *F* (1, 21) = 0.7579, *P* = 0.3938; main effect of phase *F* (2, 42) = 59.28, *P* < 0.0001, treatment *F* (1, 21) = 272.5, *P* < 0.0001, genotype *F* (1, 21) = 1.303, *P* = 0.2666; Holm-Sidak’s multiple comparisons: CNO vs Washout 1, *P* < 0.0001, CNO vs Washout 2, *P* < 0.0001, Washout 1 vs Washout 2, *P* = 0.3609). (N = 13 TH-Cre^hM3Dq^ and 10 WT^hM3Dq^ mice. Two-way interaction, *^####^P <* 0.0001, *^##^P <* 0.01, *^#^P <* 0.05; main effect of genotype, *****P <* 0.0001; Holm-Sidak’s multiple comparisons, ^σσσσ^*P <* 0.0001, ^σσ^*P <* 0.01, ^ns^*P* = not significant.)

**Additional Fig. 2 Cumulative increases in CNO-induced hyperlocomotor response in TH-Cre^hM3D(Gq)^ mice: horizontal distance traveled between 15- and 60-min post-injection period**

Analysis of distance traveled between the 15- and 60-min post-injection period between P22 and P31 shows progressive increases in CNO-induced hyperlocomotor response in TH-Cre^hM3D(Gq)^ mice compared to WT^hM3D(Gq)^ controls by two-way RM ANOVA (day x genotype interaction *F* (4, 84) = 36.18, ^####^*P* < 0.0001, genotype *F* (1, 21) = 92.52, *****P* < 0.0001, day *F* (4, 84) = 23.74, *P* < 0.0001; Holm-Sidak’s multiple comparisons: TH-Cre^hM3D(Gq)^ vs WT^hM3D(Gq)^ Day 7, ^δδ^*P* = 0.0027; Day 10, ^δδδδ^*P* < 0.0001; Day 13, ^δδδδ^*P* < 0.0001; Day 16, ^δδδδ^*P* < 0.0001; TH-Cre^hM3D(Gq)^ Day 7 vs 10, *P* = 0.0003; Day 10 vs 13, *P* < 0.0001; Day 13 vs 16, *P* = 0.0281). No genotype difference in horizontal distance is observed on the saline test day (P15) (Holm-Sidak’s multiple comparisons, Day 15, ^ns^*P* = 0.2807). N = 13 TH-Cre^hM3Dq^ and 10 WT^hM3Dq^ mice.

**Additional Fig. 3 Adolescent CNO administration has no impact on basal stereotypic behavior in early adolescent TH-Cre^hM3D(Gq)^ mice compared to WT^hM3D(Gq)^ controls**

Mice were tracked for shuffling, licking, and sniffing-like stereotypies induced by CNO 24h following each of the initial two open-field locomotor tests in the CNO phase on P23 and P26 (Experimental Days 8 and 11). Three-way ANOVA of stereotypic behavior showed that there were no main effects of day (*F* (1, 21) = 3.395, *P* = 0.0796), genotype (*F* (1, 21) = 0.03116, *P* = 0.8616), or time (*F* 1, 21) = 2.698, *P* = 0.1153), no three-way interaction (*F* (1, 21) = 1.174, ^ns^*P* = 0.2908), day-by-genotype interaction (*F* (1, 21) = 0.1785, *P* = 0.6770), or time-by-genotype interaction (*F* (1, 21) = 0.3975, *P* = 0.5352). (N = 13 TH-Cre^hM3Dq^ and 10 WT^hM3Dq^ mice. Three-way interaction, ^ns^*P*, not significant.)

**Additional Table 1**

| Exp, Figure, and Age | Depende-nt measures | Mice/  group | Statistical tests | Interacti-on and Effects | DF | t or F-value | P-value |
| --- | --- | --- | --- | --- | --- | --- | --- |
| Locomotion  Pre-inj  Fig. 1b  P22 | Horizontal distance (cm) | 13 TH-Cre  10 WT | 2-way RM  ANOVA  Curve-fit analysis | Time x Genotype interaction  Genotype  Genotype | (5,105)  (1,21)  (4,130) | 0.9713  0.9269  0.6876 | =0.4389  =0.3466  =0.6018 |
| Locomotion  Post-inj  (CNO-induced)  Fig. 1b  P22 | Horizontal distance (cm) | 13 TH-Cre  10 WT | 2-way RM  ANOVA | Time x Genotype interaction  Genotype | (11,231)  (1,21) | 4.805  14.92 | <0.0001  =0.0009 |
| Locomotion  Pre-inj  Fig. 1c  P25 | Horizontal distance (cm) | 13 TH-Cre  10 WT | 2-way RM  ANOVA  Curve-fit analysis | Time x Genotype interaction  Genotype  Genotype | (5,105)  (1,21)  (4,130) | 0.3146  0.2418  0.3814 | =0.9033  =0.6280  =0.8216 |
| Locomotion  Post-inj  (CNO-induced)  Fig. 1c  P25 | Horizontal distance (cm) | 13 TH-Cre  10 WT | 2-way RM  ANOVA | Time x Genotype interaction  Genotype | (11,231)  (1,21) | 12.5  44.33 | <0.0001  <0.0001 |
| Locomotion  Pre-inj  Fig. 1d  P28 | Horizontal distance (cm) | 13 TH-Cre  10 WT | 2-way RM  ANOVA  Curve-fit analysis | Time x Genotype interaction  Genotype  Genotype | (5,105)  (1,21)  (4,130) | 1.082  1.073  1.399 | =0.3748  =0.3120  =0.2379 |
| Locomotion  Post-inj  (CNO-induced)  Fig. 1d  P28 | Horizontal distance (cm) | 13 TH-Cre  10 WT | 2-way RM  ANOVA | Time x Genotype interaction  Genotype | (11,231)  (1,21) | 23.56  86.92 | <0.0001  <0.0001 |
| Locomotion  Pre-inj  Fig. 1e  P30 | Horizontal distance (cm) | 13 TH-Cre  10 WT | 2-way RM  ANOVA  Curve-fit analysis | Time x Genotype interaction  Genotype  Genotype | (5,105)  (1,21)  (4,130) | 1.376  0.03834  0.4786 | =0.2392  =0.8467  =0.7514 |
| Locomotion  Post-inj  (Saline-induced)  Fig. 1e  P30 | Horizontal distance (cm) | 13 TH-Cre  10 WT | 2-way RM  ANOVA | Time x Genotype interaction  Genotype | (11,231)  (1,21) | 1.458  0.7870 | =0.1487  =0.3851 |
| Locomotion  Pre-inj  Fig. 1f  P31 | Horizontal distance (cm) | 13 TH-Cre  10 WT | 2-way RM  ANOVA  Curve-fit analysis | Time x Genotype interaction  Genotype  Genotype | (5,105)  (1,21)  (4,130) | 2.088  0.6873  1.088 | =0.0726  =0.7957  =0.3653 |
| Locomotion  Post-inj  (CNO-induced)  Fig. 1f  P31 | Horizontal distance (cm) | 13 TH-Cre  10 WT | 2-way RM  ANOVA | Time x Genotype interaction  Genotype | (11,231)  (1,21) | 30.21  99.36 | <0.0001  <0.0001 |
| Locomotion  Pre-inj  Fig. 1g  P32 | Horizontal distance (cm) | 13 TH-Cre  10 WT | 2-way RM  ANOVA  Curve-fit analysis | Time x Genotype interaction  Genotype  Genotype | (5,105)  (1,21)  (4,130) | 1.129  0.05298  0.4148 | =0.3497  =0.8202  =0.7978 |
| Locomotion  Post-inj  (Saline-induced)  Fig. 1g  P32 | Horizontal distance (cm) | 13 TH-Cre  10 WT | 2-way RM  ANOVA | Time x Genotype interaction  Genotype | (11,231)  (1,21) | 0.3316  0.7710 | =0.9781  =0.3898 |
| Locomotion  Pre-inj  Fig. 1h  P33 | Horizontal distance (cm) | 13 TH-Cre  9 WT | 2-way RM  ANOVA  Curve-fit analysis | Time x Genotype interaction  Genotype  Genotype | (5,100)  (1,20)  (4,124) | 0.7470  2.726  3.408 | =0.5902  =0.1144  =0.0111 |
| Locomotion  Post-inj  (AMPH-induced)  Fig. 1h  P33 | Horizontal distance (cm) | 13 TH-Cre  9 WT | 2-way RM  ANOVA  Curve-fit analysis | Time x Genotype interaction  Genotype  Genotype | (11,220)  (1,20)  (4,256) | 0.9762  2.850  7.248 | =0.4690  =0.1069  <0.0001 |
| Stereotypy  (AMPH-induced)  Fig. 1i  P46-47 | Stereotypic movements  (% time) | 13 TH-Cre  10 WT | 3-way RM ANOVA | Treatment x Genotype interaction  Treatment  Genotype | (1,21)  (1,21)  (1,21) | 7.043  46.89  8.726 | =0.0210  <0.0001  =0.0121 |
| Locomotion  Pre-inj  Fig. 1j  P74 | Horizontal distance (cm) | 13 TH-Cre  10 WT | 2-way RM  ANOVA  Curve-fit analysis | Time x Genotype interaction  Genotype  Genotype | (5,105)  (1,21)  (4,130) | 1.579  0.4315  0.6793 | =0.1725  =0.5184  =0.6075 |
| Locomotion  Post-inj  (Saline-induced)  Fig. 1j  P74 | Horizontal distance (cm) | 13 TH-Cre  10 WT | 2-way RM  ANOVA | Time x Genotype interaction  Genotype | (11,231)  (1,21) | 1.028  1.872 | =0.4222  =0.1857 |
| Locomotion  Pre-inj  Fig. 1k  P75 | Horizontal distance (cm) | 13 TH-Cre  10 WT | 2-way RM  ANOVA  Curve-fit analysis | Time x Genotype interaction  Genotype  Genotype | (5,105)  (1,21)  (4,130) | 0.6857  0.9407  1.068 | =0.6353  =0.3431  =0.3751 |
| Locomotion  Post-inj  (AMPH-induced)  Fig. 1k  P75 | Horizontal distance (cm) | 13 TH-Cre  10 WT | 2-way RM  ANOVA  Curve-fit analysis | Time x Genotype interaction  Genotype  Genotype | (11,231)  (1,21)  (4,268) | 0.8739  0.3590  1.283 | =0.5667  =0.5555  =0.2770 |
| Stereotypy  (AMPH-induced)  Fig. 1l  P81-82 | Stereotypic movements  (% time) | 13 TH-Cre  10 WT | 3-way RM ANOVA | Treatment x Genotype interaction  Treatment  Genotype | (1,21)  (1,21)  (1,21) | 0.4234  67.94  0.3654 | 0.5220  <0.0001  0.5517 |
| Locomotion  Pre-inj  Fig. 1m  P109 | Horizontal distance (cm) | 13 TH-Cre  10 WT | 2-way RM  ANOVA  Curve-fit analysis | Time x Genotype interaction  Genotype  Genotype | (5,105)  (1,21)  (4,130) | 1.137  1.666  2.340 | =0.3457  =0.2109  =0.0585 |
| Locomotion  Post-inj  (Saline-induced)  Fig. 1m  P109 | Horizontal distance (cm) | 13 TH-Cre  10 WT | 2-way RM  ANOVA | Time x Genotype interaction  Genotype | (11,231)  (1,21) | 1.701  0.4543 | =0.0740  =0.5076 |
| Locomotion  Pre-inj  Figure 1n  P110 | Horizontal distance (cm) | 13 TH-Cre  10 WT | 2-way RM  ANOVA  Curve-fit analysis | Time x Genotype interaction  Genotype  Genotype | (5,105)  (1,21)  (4,130) | 0.1124  0.3464  0.4386 | =0.9894  =0.5625  =0.7805 |
| Locomotion  Post-inj  (AMPH-induced)  Fig. 1n  P110 | Horizontal distance (cm) | 13 TH-Cre  10 WT | 2-way RM  ANOVA  Curve-fit analysis | Time x Genotype interaction  Genotype  Genotype | (11,231)  (1,21)  (4, 268) | 0.3745  0.2399  0.7505 | =0.9649  =0.6294  =0.5584 |
| Stereotypy  (AMPH-induced)  Fig. 1o  P116-117 | Stereotypic movements  (% time) | 13 TH-Cre  10 WT | 3-way RM ANOVA | Treatment x Genotype interaction  Treatment  Genotype | (1,21)  (1,21)  (1,21) | 0.1008  304.4  0.2972 | 0.7539  <0.0001  0.5911 |
| Locomotion  Pre-inj  Fig. 1p | Horizontal distance (cm) | 13 TH-Cre  10 WT | LMM | Day x Genotype interaction  Genotype | estimate+/-SE = -85.248  +/-256.121  estimate+/-SE = 388.864  +/-2905.141 | .333  -.134 | =0.740  =0.894 |
| Locomotion  Pre-inj  Fig. 1q | Horizontal distance (cm) | 13 TH-Cre  10 WT | LMM | Phase x Genotype interaction  Genotype | estimate+/-SE = -2312.806  +/-2554.655  estimate+/-SE =-153.805  +/-4542.152 | .905  -.034 | =0.369  =0.973 |
| Locomotion  Post-inj  (Saline-induced)  Fig. 1r | Horizontal distance (cm) | 13 TH-Cre  10 WT | LMM | Phase x Genotype interaction  Genotype | estimate+/-SE = -8981.892  +/-7832.608  estimate+/-SE =-10896.63  +/-14917.2 | 1.147  -.730 | =0.256  =0.468 |
| Locomotion  Post-inj  (AMPH-induced)  Fig. 1r | Horizontal distance (cm) | 13 TH-Cre  10 WT | LMM | Phase x Genotype interaction  Genotype | estimate+/-SE = -5601.149  +/-24407.73  estimate+/-SE =-34451.31+/-52226.61 | -.229  .660 | =0.819  =0.513 |
| Stereotypy  (Saline-induced) Figure 1s | Stereotypic movements  (% time) | 13 TH-Cre  10 WT | LMM | Phase x Genotype interaction  Genotype | estimate+/-SE = -0.990+/-1.238  estimate+/-SE = 1.678+/-2.837 | -0.800  0.592 | =0.428  =0.557 |
| Stereotypy  (AMPH-induced)  Fig. 1s | Stereotypic movements  (% time) | 13 TH-Cre  10 WT | LMM | Phase x Genotype interaction  Genotype | estimate+/-SE = -17.731+/-6.740  estimate+/-SE = 47.450+/-16.819 | -2.631  2.821 | =0.012  =0.007 |

**Additional Table 1. Summary of statistical tests and outcomes for Fig. 1**

The table presents the dependent measures, number of animals used per group, the statistical tests employed, and the Degrees of Freedom (DF), t and F-values, and P-values for each experiment. *Pre-inj,* Pre-injection (baseline); *Post-inj,* Post-injection; *CNO,* clozapine-N-oxide; *AMPH*, Amphetamine; *LMM,* Linear Mixed Model

**Additional Methods**

**Mice**

All experimental studies were approved by the New York State Psychiatric Institute Institutional Animal Care and Use Committee (IACUC) in accordance with the NIH’s Guide for the Care and Use of Laboratory Animals. TH-Cre mice (1) (Strain No. 008601, The Jackson Laboratory) were crossed with C57BL/6 J wildtype (WT) mice to obtain heterozygous TH-Cre and littermate control WT animals. All experiments were conducted blind to genotype. Mice were group housed under a 12h light/dark cycle in a temperature-controlled environment, with food and water available *ad libitum*. A total of 13 TH-Cre^hM3D(Gq)^ (9 males/4 females) and 10 WT^hM3D(Gq)^ (5 males/5 females) mice were evaluated for baseline and drug-induced locomotor and stereotypic behaviors. We previously reported no significant differences in baseline and AMPH-induced behaviors, or an effect of sex on these behaviors, in TH-Cre mice relative to WT mice (2).

**Experimental timeline**

See Main Fig. 1a for schematic of experimental timeline. Virus injections took place on postnatal day 1 (P1) [termed Experimental Day (-)14]. Pups were allowed to recover for 14 days before commencement of 1x daily intraperitoneal (IP) 1.0 mg/kg CNO treatment on P15 (Day 0). Behavioral experiments commenced on P22 (Day 7).

During CNO phase (P15-47/Day 0-32), CNO was administered to mice in the clear open-field and stereotypy shoebox chambers. CNO was administered at the end of the behavioral session on saline and AMPH test days in home cages. On all other non-test days, CNO was administered to mice in home cages.

During CNO phase, mice were evaluated for (a) CNO-induced locomotor response every three days on Days 7, 10, 13 and 16, (b) saline-induced locomotion on Days 15 and 17, and (c) AMPH-induced locomotor response on Day 18. CNO-induced stereotypic behavior response was conducted 24h following each of the initial two open-field CNO tests on Day 8 and 11. AMPH-induced stereotypic response was evaluated 2 weeks following the open-field AMPH test on Days 31-32.

To evaluate recovery of behavioral response, mice were again evaluated for saline and AMPH-induced locomotion and stereotypic behavior one month and two months after stopping CNO, during Washout phase 1 (P48-82/ Days 33-67) and Washout phase 2 (P83-117/ Days 68-102).

**Virus injections**

P1 pups were anesthetized by hypothermia and placed into a motorized stereotactic apparatus (Stoelting). A glass capillary attached to a Nanoject (Drummond) was slowly lowered into the midbrain (from lambda, AP -0.15, ML ± 0.20, DV -3.0mm) and 0.20 ul of undiluted AAV-hSyn-DIO-hM3D(Gq)-mCherry (Addgene, Cat No. 44361-AAV5, titer ≥ 7 × 10^12^vg/mL) was delivered at a flow rate of 0.1 ul/min. The glass capillary was left in place for 10 min to facilitate diffusion into the brain tissue. After 10 min, the capillary was slowly retracted, and the procedure was repeated for the opposite hemisphere. Following surgery, pups were promptly placed onto a heating pad (37 °C). Pups were returned to the dam upon regaining of consciousness.

**Histology**

Mice were transcardially perfused with ice-cold 4% (wt/vol) paraformaldehyde (PFA) in phosphate-buffered saline (PBS). Brains were extracted, post-fixed in PFA for 6h and then cryoprotected in ascending concentrations (10, 20 and 30%) of sucrose (in PBS) at 4°C. 40 um thick coronal sections spanning the midbrain region were cut on a cryostat and collected in PBS. Sections were washed in PBS three times and blocked with 10% normal goat serum and 0.3% Triton X-100 in PBS for 2h at room temperature (RT). Sections were incubated overnight at 4°C in 10% normal goat serum and 0.3% Triton-X with primary anti-TH (dilution 1:1000, mouse monoclonal, Sigma, T2928) and anti-RFP antibody (dilution 1:500, rabbit polyclonal, Rockland, 600-401-379) in PBS. Sections were incubated with Alexa-conjugated secondary antibodies (dilution 1:500, goat anti-mouse, Alexa-Fluor 488, Life Technologies, A-11029; goat anti-rabbit, Alexa-Fluor 594, Abcam, ab150080) for 1h at RT. After three more steps of washing in PBS, sections were mounted on a glass slide and coverslipped. Sections were visualized using a laser confocal microscope (TCS SP8, Leica Microsystems, Wetzlar, Germany) operated through the LAS X software v 3.5.5.19976 (Leica). Images were acquired with a 63x objective.

**Behavior**

**Open-field locomotion**

Mice were placed in a clear open-field chamber (40.6 cm L × 40.6 cm W, SmartFrame Open Field System, Kinder Scientific) fitted with 32 infrared photo-beams and connected to the Motor Monitor Software (Kinder Scientific). Following an initial exploratory period of 30 min, mice were administered intraperitoneal (IP) dose of 1.0 mg/kg CNO, 0.9% saline, or 3.0 mg/kg AMPH, depending on the test session, and locomotor activity was monitored over the next 60 min. Locomotion was recorded under bright ambient light conditions.

**Stereotypic behavior**

Stereotypic behavior was assessed in clear shoebox cages (30.5 cm L × 19.7 cm W × 16.5 cm H). Following 30 min of acclimation, mice were administered an IP dose of 1.0 mg/kg CNO, saline, or 8.0 mg/kg AMPH, depending on the test session. Mouse behavior was recorded using a video camera (Sony Handycam Flash Memory Camcorder, HDRCX405/B) for 2 mins each at 50- and 80-min post-drug administration (termed as T50 and T80). Two trained video observers, blind to genotype and treatment, manually recorded the time spent in stationary shuffling, licking and sniffing-like stereotypy, as previously described (3).

**Drugs**

Clozapine-N-oxide (CNO) (Tocris Bioscience) was dissolved in 0.25% DMSO / 0.9% sterile saline. D**-**amphetamine (AMPH) (Sigma-Aldrich) was dissolved in 0.9% sterile saline. All drugs were administered IP at a volume of 10.0 ml/kg.

**Statistical analysis**

Data was analyzed with GraphPad Prism (version 10.2.0, GraphPad Software, San Diego, CA, USA) and SPSS (IBM SPSS Statistics for MacOS, Version 28.0.1.1). Horizontal locomotor activity was collected in 5-min bins and analyzed using two-way RM ANOVA and non-linear curve-fit. Area Under the Curve (AUC) was calculated and compared using three-way or two-way RM ANOVA, and Linear Mixed Model (LMM) analysis. Additionally, horizontal distance traveled between the 15- and 60-min post-CNO injection period was averaged and compared using two-way RM ANOVA. Stereotypy was analyzed using three-way ANOVA and LMM. For LMM, individual animals defined the subject variables, AUC and % time spent in stereotypy as the dependent variables, genotype as the independent factor, and day and time as the continuous covariate. Random effects associated with the intercept and the slope for each subject were included in the model. Akaike’s Information Criterion (AIC) was used to determine the covariance structure and revealed that the model with the best fit had a Scaled Identity covariance matrix. All data are reported as the mean ± standard error of the mean (SEM).

**References**

1. Savitt JM, Jang SS, Mu W, Dawson VL, Dawson TM. Bcl-x is required for proper development of the mouse substantia nigra. J Neurosci. 2005;25(29):6721-8.

2. Chohan MO, Esses S, Haft J, Ahmari SE, Veenstra-VanderWeele J. Altered baseline and amphetamine-mediated behavioral profiles in dopamine transporter Cre (DAT-Ires-Cre) mice compared to tyrosine hydroxylase Cre (TH-Cre) mice. Psychopharmacology (Berl). 2020;237(12):3553-68.

3. Chohan MO, Fein H, Mirro S, O’Reilly KC, Veenstra-VanderWeele J. Repeated chemogenetic activation of dopaminergic neurons induces reversible changes in baseline and amphetamine-induced behaviors. Psychopharmacology. 2023;240(12):2545-60.
